# Somatic alterations and mutational burden are potential predictive factors for metachronous development of early gastric cancer

**DOI:** 10.1038/s41598-020-79195-0

**Published:** 2020-12-16

**Authors:** Kazuhiro Sakuta, Yu Sasaki, Yasuhiko Abe, Hidenori Sato, Masakuni Shoji, Takao Yaoita, Makoto Yagi, Naoko Mizumoto, Yusuke Onozato, Takashi Kon, Ayumi Koseki, Sonoko Sato, Ryoko Murakami, Yuki Miyano, Yoshiyuki Ueno

**Affiliations:** 1 Department of Gastroenterology, Faculty of Medicine, Yamagata University, 2-2-2 Iida-Nishi, Yamagata, 990-9585 Japan; 2 Division of Endoscopy, Yamagata University Hospital, 2-2-2 Iida-Nishi, Yamagata, 990-9585 Japan; 3 Genomic Information Analysis Unit, Department of Genomic Cohort Research, Yamagata University Faculty of Medicine, 2-2-2 Iida-Nishi, Yamagata, 990-9585 Japan

**Keywords:** Gastroenterology, Medical research, Molecular medicine, Oncology, Risk factors

## Abstract

The risk of developing metachronous gastric cancer (MGC) following curative endoscopic submucosal dissection (ESD) of early gastric cancer (EGC) remains even after eradicating *Helicobacter pylori* (HP) successfully. We screened initial EGC and adjacent non-cancerous mucosa ESD-resected specimens for somatic variants of 409 cancer-related genes, assessing their mutational burden (MB) to predict molecular markers for metachronous post-ESD development. We compared variants between ten patients diagnosed with MGC more than 3 years after ESD and ten age-matched patients who did not have MGC developments after successful HP eradication. We found no significant background differences between the two groups. In adjacent non-cancerous mucosa, the MB tended to be higher in the patients with metachronous developments than in the others. Somatic genomic alterations of *RECQL4*, *JAK3*, *ARID1A*, and *MAGI1* genes were significantly associated with MGC development. The criteria including both the MB and their variants, which had potential significant values for predicting MGC. In conclusion, combined of assessing specific somatic variants and MB may be useful for predicting MGC development. This study included a limited number of subjects; however, our novel findings may encourage further exploration of the significance of the molecular features of EGC that predict MGC development, thereby promoting focused follow-up strategies and helping elucidate the mechanisms.

## Introduction

Gastric cancer is the fifth most frequently diagnosed cancer and the third leading cause of death worldwide^1^. Endoscopic submucosal dissection (ESD) is a well-established procedure, showing good prognoses after treatment of early stage neoplasms in the gastrointestinal tract, including early gastric cancer (EGC)^2,3^. However, the risk of metachronous gastric cancers (MGCs) development following curative ESD of EGCs needs to be addressed properly^4^. The MGC incidence rate is approximately 2.7–15.6% after endoscopic resection^5^. Although successfully eradicating *Helicobacter pylori* (HP) has a significant preventive impact on MGC development^6–8^, the risk remains present. Therefore, patients who undergo curative ESDs receive standard follow-up EGDs, at least annually, throughout their lives (even after successful HP eradication) to detect any developing MGCs at an early stage^9,10^. A predictive marker for identifying patients with an increased risk of MGC is needed to establish a proper, efficient follow-up strategy.

Reports have shown that older people, men, current smokers, patients with multiple initial EGCs, or those with severe gastric mucosal atrophy have an increased risk of MGC^5^. Expression of CD44 variant 9 (CD44v9), a major adhesion molecule for the extracellular matrix, and a functional cancer stem cell marker in primary EGC lesions leads to a higher MGC rate than it would in CD44v9-negative tumors^11^. In addition, the non-cancerous gastric mucosa’s methylation level in gastric cancer (GC) patients, especially in the tumor-suppressor *miR-124a-3*, was significantly associated with an increased risk of developing MGC by a multicenter, prospective cohort study^12,13^. These findings suggest that altering the normal gastric mucosa or initial EGCs may be markers for MGC occurrence.

Like most solid epithelial cancers, GCs are often characterized by somatically-acquired mutations in various genes^14^. In fact, the Cancer Genome Atlas (TCGA) project provides a landmark in the molecular characterization of mainly advanced GC and identification of four subtypes^15^, and is considered a potential roadmap for patient stratification and trials of targeted therapies. However, studies on EGCs’ genetic features, including those of the normal gastric mucosa, are scarce, and whether these genetic features may predict MGC development remains unclear. Therefore, we surveyed the somatic variants present in ESD-resected specimens obtained from patients with metachronous developments. We used next-generation sequencing (NGS) panel analyses on both the initial EGC and the non-cancerous mucosa specimens. Our results suggested that alterations of specific somatic variants and mutational burden may be linked to the risk of MGC development. The further analysis of these alterations, using a large number of patients, may help to estimate the risk of MGC and elucidate the mechanisms.

## Results

### Clinical characteristics of the patients

**Figure 1:**
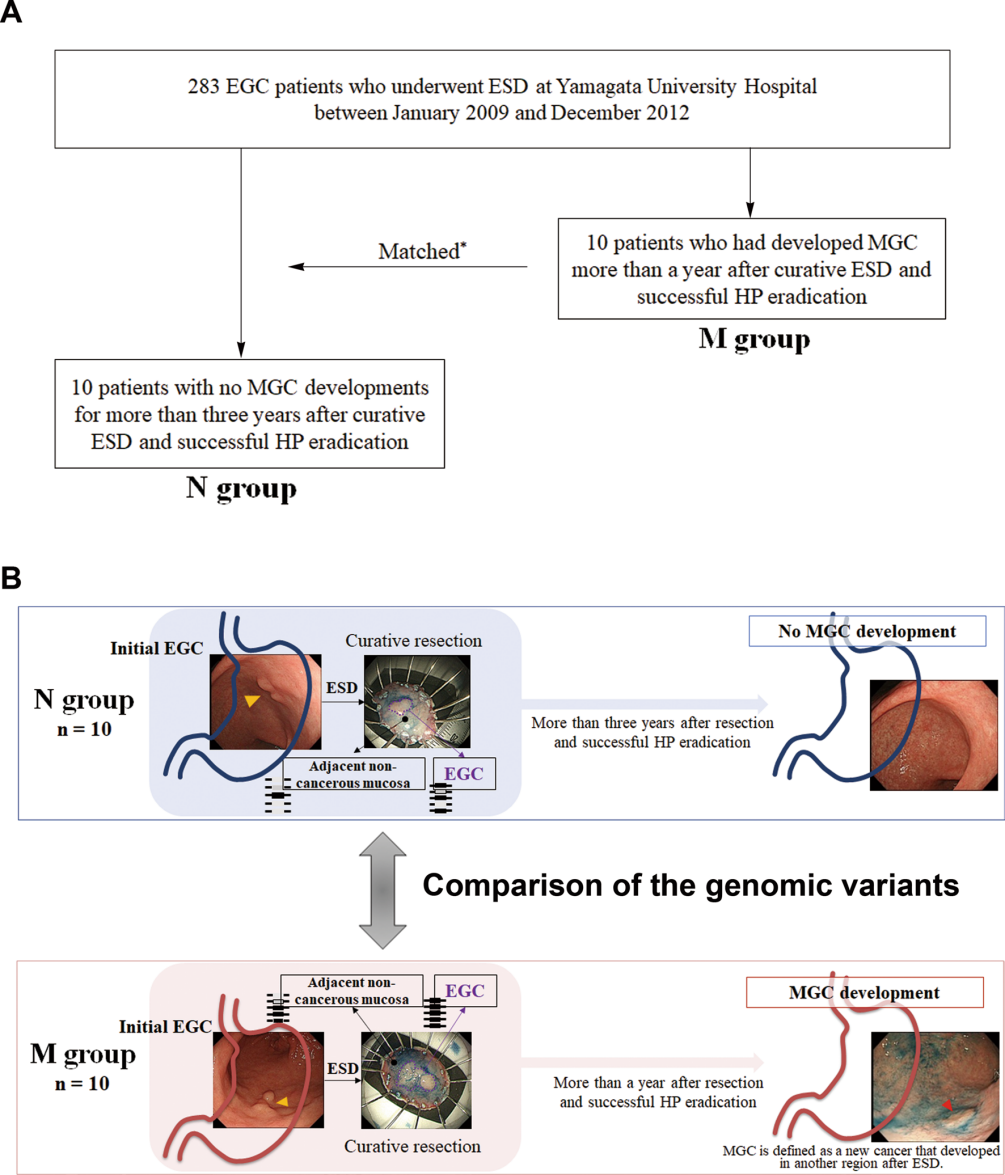
Study design. Among 283 EGC patients who underwent ESD at the Yamagata University Hospital between January 2009 and December 2012 (**A**), we selected ten patients who had developed MGC more than a year after curative ESD and successful HP eradication (the M group). We then selected ten patients with no MGC developments for more than 3 years after ESD and successful HP eradication (the N group). The somatic variants in initial EGC and adjacent non-cancerous mucosa were compared between the samples obtained from the M group and those obtained from the N group (**B**). We used the term “initial EGC” to differentiate the condition from MGC, which is also called “secondary cancer”. *The patients in the N group matched the ten patients in the M group in terms of age, gender, location, size, and histological type of the initial EGC. *EGC* early gastric cancer, *ESD* endoscopic submucosal dissection, *MGC* metachronous gastric cancer, *HP* *Helicobacter pylori*.

The study design was shown in Fig. 1. We found no significant differences between the non-MGC development (N) group and the metachronous development (M) group in terms of men/women ratios, alcohol consumption, current smoker rates, tumor locations, macroscopic types, gastric mucosal atrophy degrees, the presence of intestinal metaplasia, and age, body mass index, or maximum size of tumor means (Table 1, Suppl. Table 1). All initial EGCs were of the intestinal type and were confined to the mucosa.

**Table 1:** Clinical characteristics at the time of initial endoscopic treatment.

	N group N = 10	M group N = 10	*p*
Age, years	66.0 ± 6.0	66.5 ± 6.7	Matched
Male	8 (80)	8 (80)	Matched
BMI, kg/m^2^	23.8 ± 3.1	23.4 ± 3.7	0.90
Alcohol consumption rate	3 (30)	6 (60)	0.36
Current smoking rate	6 (60)	7 (70)	1.00
**Gastric mucosal atrophy**			Matched
Open type	10 (100)	10 (100)	
Closed type	0 (0)	0 (0)	
Intestinal metaplasia	10 (100)	10 (100)	1.00
**Tumor location**			Matched
Antrum	6 (60)	6 (60)	
Body	4 (40)	4 (40)	
Tumor size, mm	14.5 ± 6.6	17.7 ± 14.7	0.84
**Macroscopic type**			1.00
Depressed	7 (70)	7 (70)	
Elevated	3 (30)	3 (30)	
**Pathological type**			Matched
Intestinal	10 (100)	10 (100)	
Diffuse	0 (0)	0 (0)	
**Depth of invasion**			Matched
Mucosal lesion	10 (100)	10 (100)	
After successful eradication*, months	65.5 ± 17.6	38.5 ± 16.9	< 0.01

Values are expressed as means ± standard deviations or as n (%). *We selected the patients in the M group > 1 year after they had undergone curative resection and successful HP eradication; we selected the patients in the N group > 3 years after they had undergone ESD and successful HP eradication.

### Characteristics of somatic alterations

**Figure 2:**
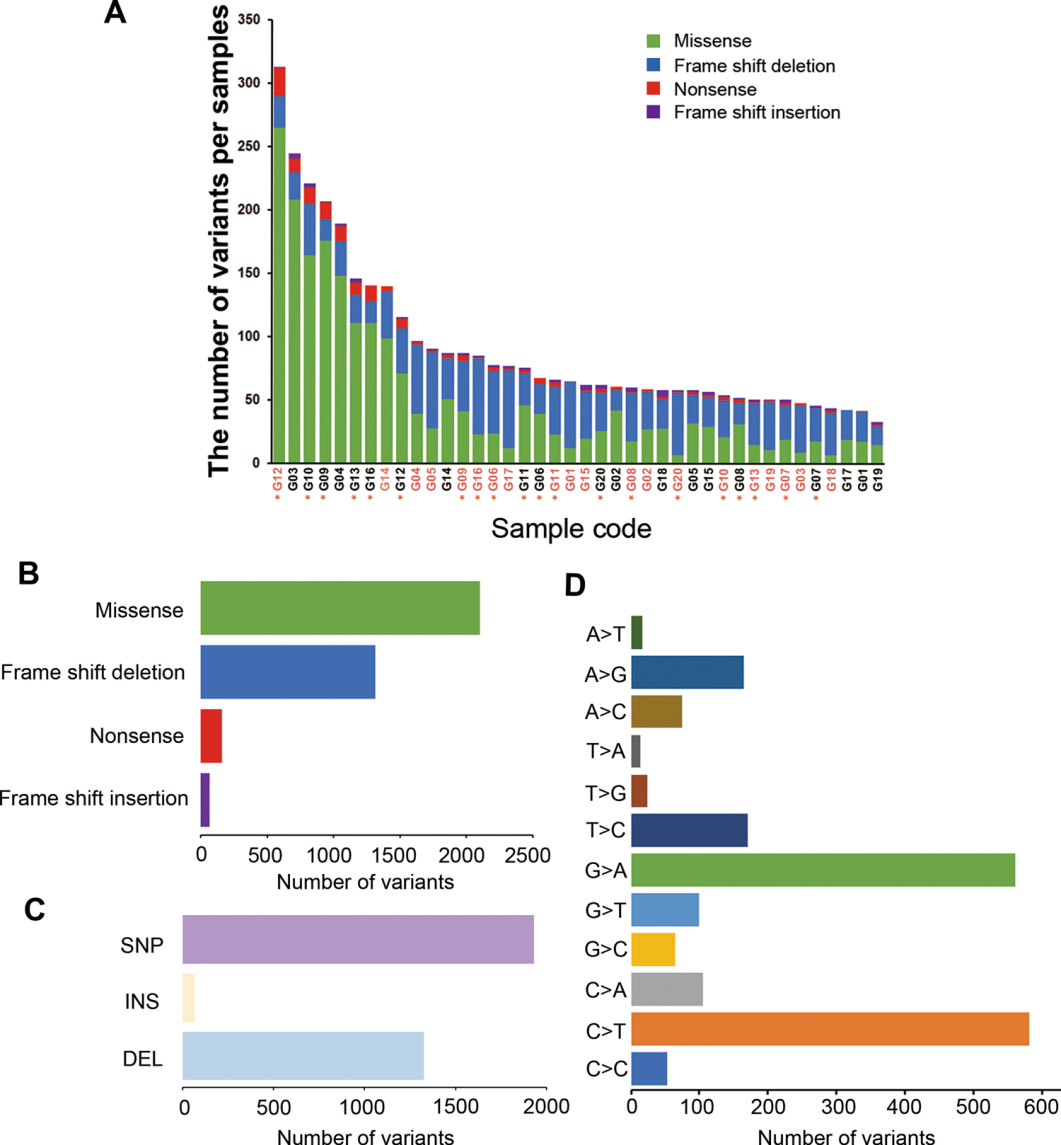
Summary of identified somatic variants. The total somatic variants in each sample, which are predicted to be protein altering, is displayed in a stacked bar plot (**A**: orange names belong to cancerous tissue, black ones belong to adjacent non-cancerous specimens; the asterisk indicates the metachronous development group). The median numbers of total somatic variants, missenses, frame shift deletions, nonsense, and frame shift insertions are 63.5 (IQR, 52.5–95.5), 28 (IQR, 18–61), 30 (IQR, 23.5–39), 2 (IQR, 1–4.5), and 2 (IQR, 1–2.5), respectively. Variant types in a boxplot summarized by variant classification (**B**) and type (**C**), and by SNP class (**D**). *SNP* single nucleotide polymorphism, *INS* insertion, *DEL* deletion.

We identified 71,100 variants in the NGS panel (Suppl. Table 2 shows a complete list of all somatic variants), of which 3645 somatic variants were predicted to be protein altering (2103 missense, 1313 frame shift deletions, 162 nonsense, and 67 frame shift insertions; Fig. 2A,B). The variant load in our study stood almost in the middle of 33 landmark cohorts in the TCGA (Suppl. Fig. 1). The most common type of variant was a single nucleotide polymorphism (SNP; 58.0%, Fig. 2C), and most were C-to-T (30.2%) and G-to-A (29.1%) transitions (Fig. 2D). The median transition/transversion (Ti/Tv) ratio was 2.25 [Interquartile range (IQR), 1.11–3.00, Suppl. Fig. 2]. We restricted the next analyses to those 3645 somatic variants.

### Comparison of somatic variants between the N and the M groups considering both initial EGC and adjacent non-cancerous mucosa

**Figure 3:**
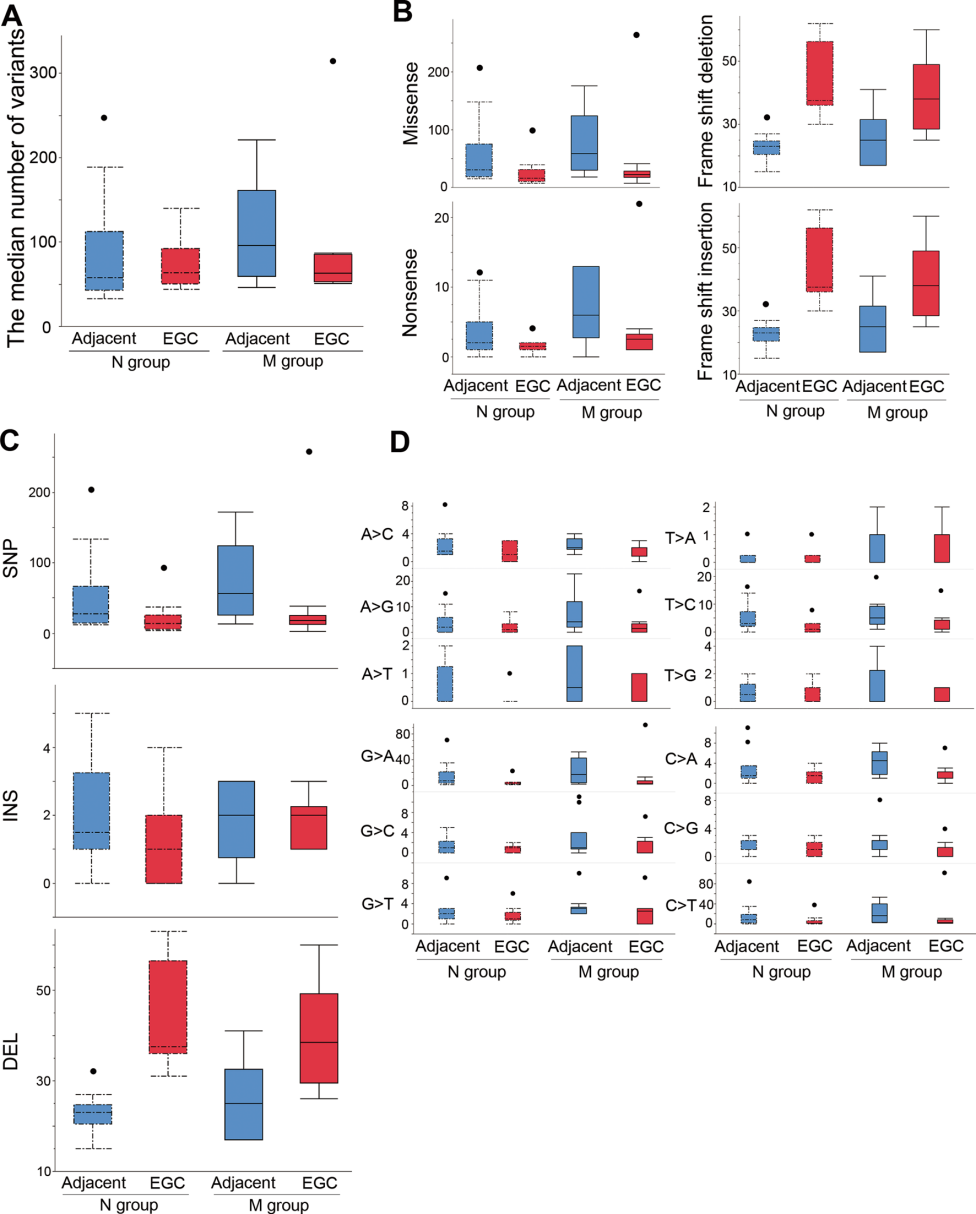
Comparison of somatic variants between the N and M groups. The median numbers of somatic variants were 58.0 (IQR, 42.7–112.5) in the adjacent non-cancerous mucosa of the N group, 63.5 (IQR, 50.2–92.5) in the initial EGC of the N group, 96.0 (IQR, 59.5–161.2) in the adjacent non-cancerous mucosa of the M group, and 63.0 (IQR, 53.2–85.5) in the initial EGC of the M group (*p* = 0.36, **A**). The classification (**B**) or type (**C**) of variants and SNP classes (**D**) were not significantly different between the groups. Box plot: the bottom and top of each box represent the 25th and 75th percentiles, respectively, and the band in the box is the median. Whiskers: the lowest datum is within the minimum, and the highest datum is still within the 1.5 IQR of the upper quartile. Values of outliers are drawn as dots. We used the Kruskal–Wallis test, with post hoc Steel–Dwass test, to evaluate the statistical differences between groups. Actual values are provided in Suppl. Table 3. *SNP* single nucleotide polymorphism, *INS* insertion, *DEL* deletion.

The median number of somatic variants was 63.5 (IQR, 52.5–95.5) in all cases. We found no significant differences in the number of somatic variants between the N group and the M groups (Fig. 3A). In both the N and the M groups, the numbers of missense variants (*p* = 0.01) and nonsense variants (*p* = 0.04) were significantly higher in the adjacent non-cancerous mucosa specimens than in the initial EGC specimens, whereas the numbers of frame shift deletions (*p* < 0.01) or insertions (*p* < 0.01) were significantly higher in the initial EGC specimens than in the adjacent non-cancerous mucosa specimens (Fig. 3B). We found no significant differences between the N and the M groups in terms of the classifications or types of variants (Fig. 3B,C). Moreover, G-to-A and C-to-T transitions were more frequent in the adjacent non-cancerous mucosa than in the initial EGC in both groups, but not significantly so (Fig. 3D).

### Mutational burden between the N and the M groups in initial EGC and adjacent non-cancerous mucosa specimens

**Figure 4:**
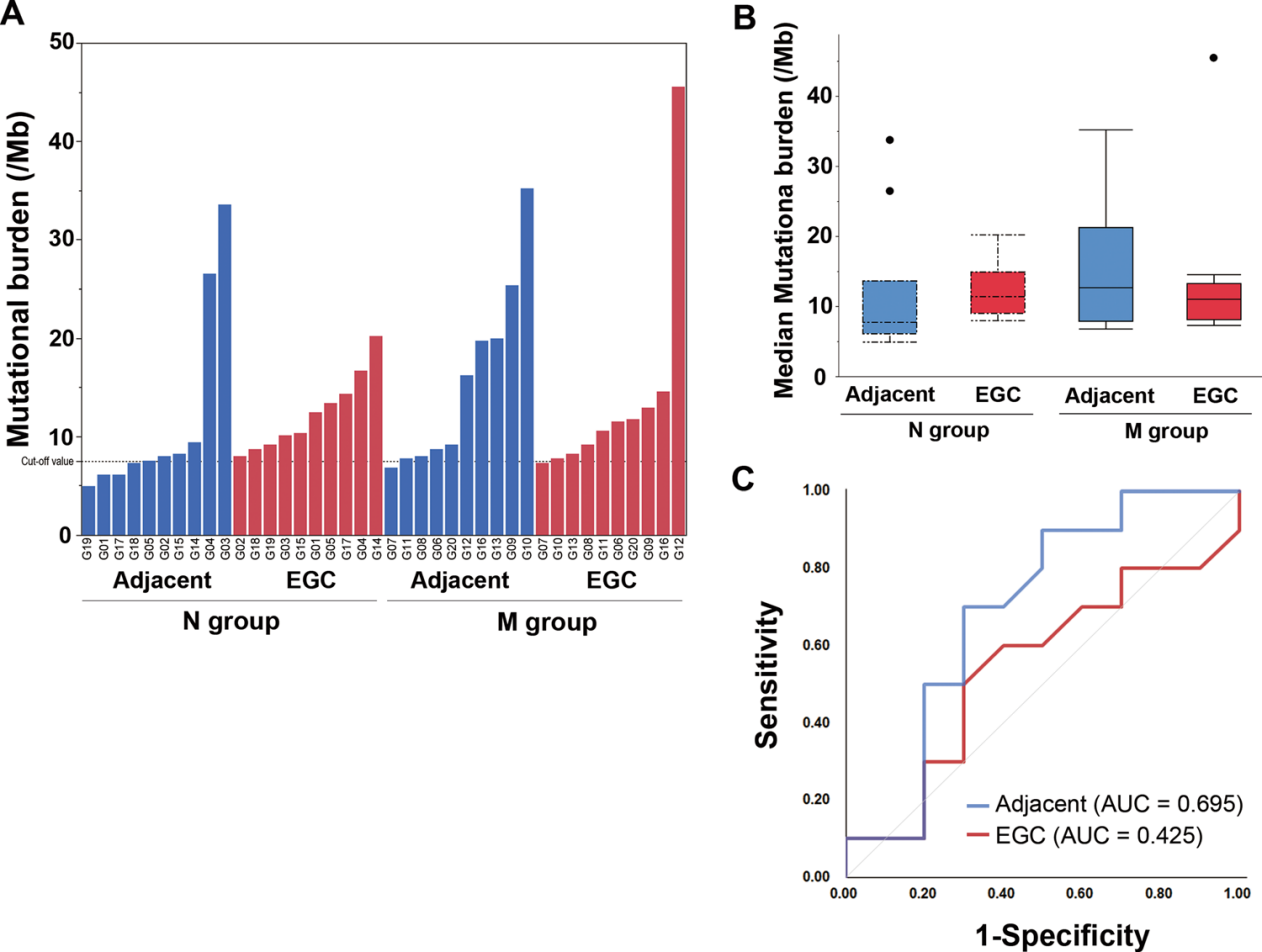
Mutational burden. MBs (**A**,**B**) tended to be higher in the M group [12.6 (7.9–21.3), in the adjacent non-cancerous mucosa, and 11.0 (8.1–13.3) in the initial EGC] than in the N group [7.7 (6.1–13.6) in the adjacent non-cancerous mucosa, 11.3 (9.0–14.9) in the initial EGC]. ROC analysis (**C**) showing that the AUC value of the MB in the adjacent, non-cancerous mucosa is higher than that in the initial EGC, and confirms 7.75 as the cutoff value for predicting metachronous developments. Box plot: the bottom and top of each box represent the 25th and 75th percentiles, respectively, and the band in the box is the median. Whiskers: the lowest datum is within the minimum, and the highest datum is still within the 1.5 IQR of the upper quartile. Values of outliers are drawn as dots. *MB* mutational burden, *EGC* early gastric cancer, *ROC* receiver operating characteristic, *AUC* area under the curve, *IQR* interquartile range.

In initial EGC specimens, the MBs were similar between the N and M groups, whereas in the adjacent non-cancerous mucosa, the MBs tended to be higher in the M group than in the N group (Fig. 4A,B). Based on our receiver operating characteristic (ROC) analysis (Fig. 4C), the area under the curve (AUC) value for MB in the adjacent non-cancerous mucosa (0.695) was higher than that of the initial EGC (0.425) for predicting metachronous developments. The calculated cutoff point was 7.75 (Suppl. Table 4).

### Specific genes in the N and M groups

**Figure 5:**
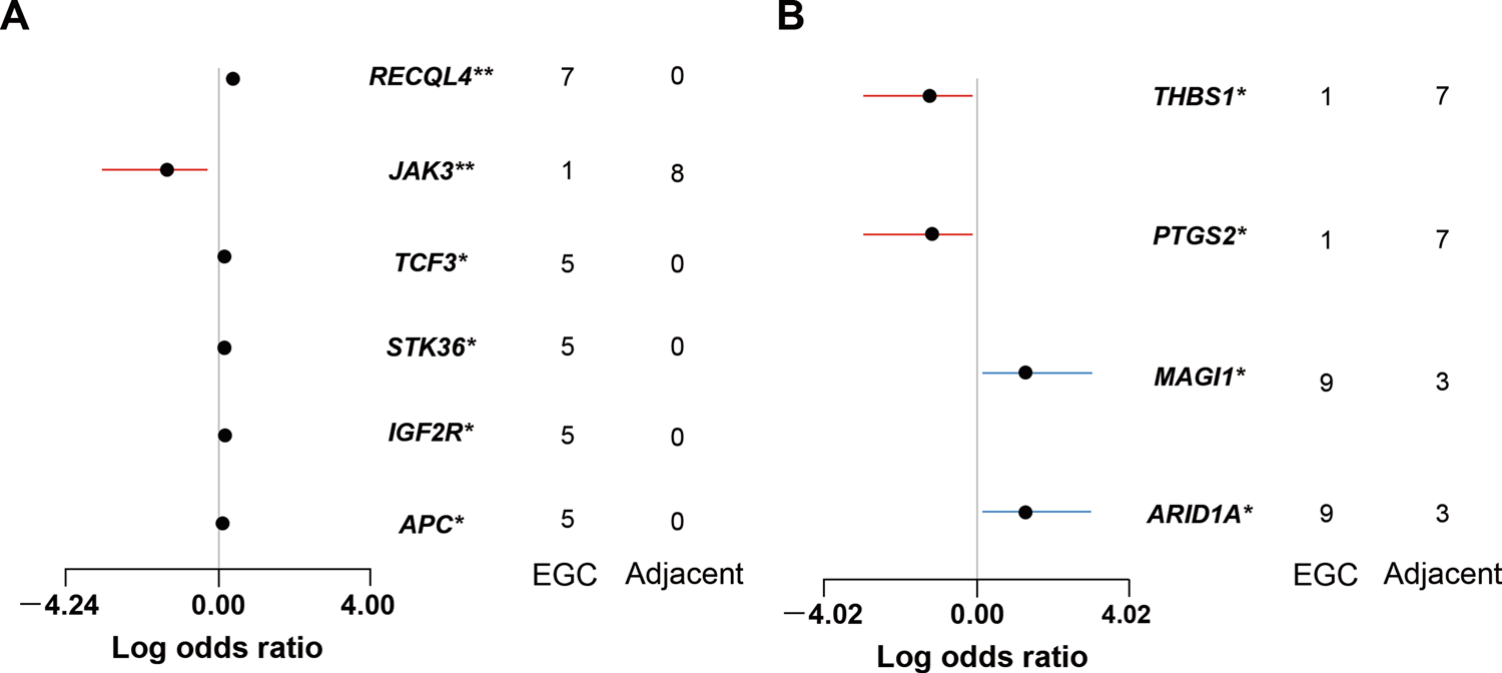
Specific genes harboring somatic variants associated with the initial EGC and the adjacent non-cancerous mucosa in the N group (**A**) or M group (**B**). We evaluated log odds ratios based on a false discovery rate < 0.3. *p < 0.05, **p < 0.01. *RECQL4* RecQ like helicase 4, *TCF3* transcription factor 3, *STK36* serine/threonine kinase 36, *IGF2R* insulin like growth factor 2 receptor, *APC* adenomatous polyposis coli, *JAK3* Janus kinase 3, *ARID1A* AT-rich interactive domain containing protein 1A, *MAGI1* membrane associated guanylate kinase WW and PDZ domain containing 1, *THBS1* thrombospondin 1, *PTGS2* prostaglandin-endoperoxide synthase 2.

We compared the genes harboring somatic variants between the initial EGC and the adjacent non-cancerous mucosa specimens in the N and M groups (Fig. 5) with a false discovery rate < 0.3. In the N group (Fig. 5A), the initial EGCs had more variants in the *RecQ like helicase 4* (*RECQL4*), *Transcription factor 3* (*TCF3*), *Serine/threonine kinase 36* (*STK36*), *Insulin like growth factor 2 receptor* (*IGF2R*), and the *Adenomatous polyposis coli* (*APC*) genes, which were absent from the adjacent non-cancerous mucosa specimens, whereas the *Janus kinase 3* (*JAK3*) gene was highly altered in the adjacent non-cancerous mucosa. In the M group (Fig. 5B), the *AT-rich interactive domain containing protein 1A* (*ARID1A*) and the *membrane associated guanylate kinase, WW and PDZ domain containing 1* (*MAGI1*) genes were highly altered in the initial EGCs, whereas the adjacent non-cancerous mucosa tended to have more variants in the *thrombospondin 1* (*THBS1*) and the *prostaglandin-endoperoxide synthase 2* (*PTGS2*) genes.

### Predictive criteria for metachronous development

**Figure 6:**
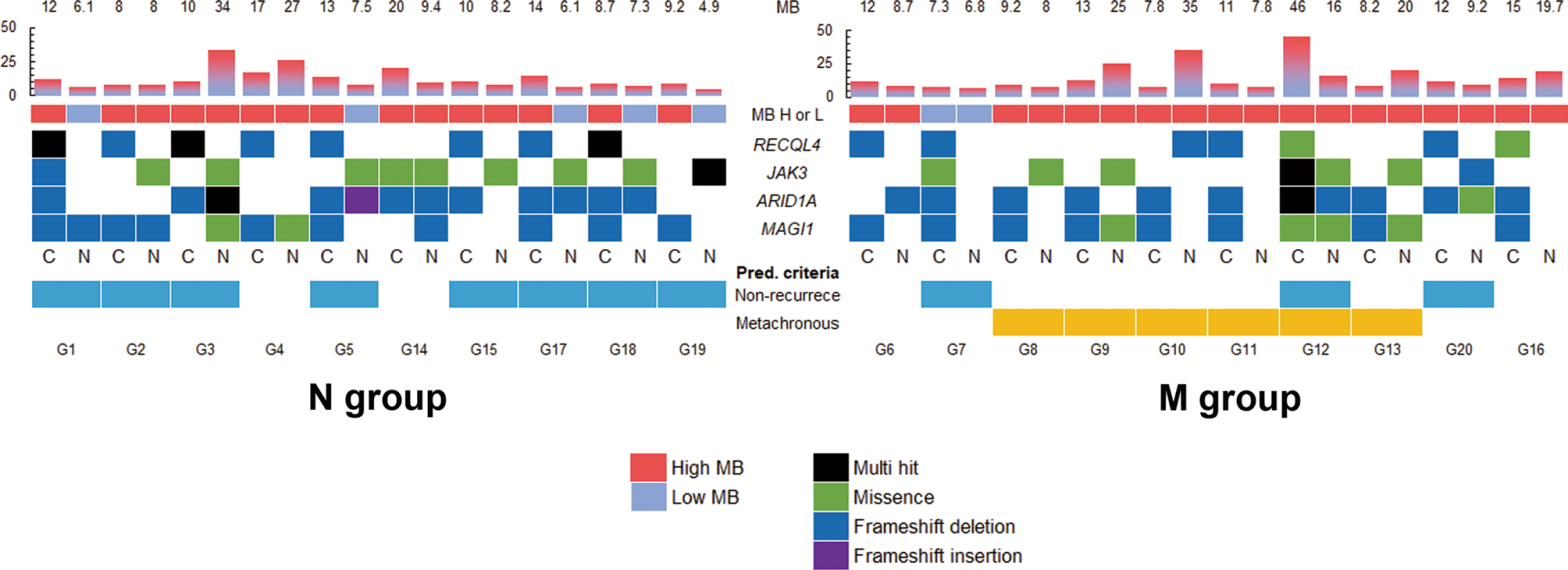
Prediction of metachronous development or non-development based on MB and specific gene variants. MB, mutational burden; H, high; L, low; *RECQL4*, *RecQ like helicase 4*; *JAK3*, *Janus kinase 3*; *ARID1A*, *AT-rich interactive domain containing protein 1A*; *MAGI1*, *membrane associated guanylate kinase WW and PDZ domain containing 1*; N, adjacent non-cancerous lesion; C, initial EGC; Pred. criteria, predictive criteria. The patients met the predictive criteria of non-development as blue whereas metachronous development as orange.

We sought to identify predictive criteria for metachronous development of gastric cancer by combining MB with specific gene variants (Fig. 6). The criteria (Table 2) for predicting non-MGC development included cases with initial EGC harboring *RECQL4* variants with *JAK3* variants or a low MB in the adjacent non-cancerous mucosa. In the N group, 70% of the patients met the criteria (*p* = 0.01). The MGC prediction criteria were cases with initial EGC harboring variants in both the *ARID1A* and *MAGI1* with high MBs in the adjacent non-cancerous mucosa. In the M group, 70% of the patients met the MGC prediction criteria, whereas none in N group fulfilled the MGC prediction criteria (*p* < 0.01).

**Table 2:** Predictive criteria for MGC development or non-development.

Prediction	Criteria			N group	M group	*p*
	Initial EGC		Adjacent non-cancerous mucosa			
Non development	*RECQL4*-variant positive	and	*JAK3* positive or low MB	7 (70)	1 (10)	0.019
Metachronous development	*ARID1A-* and *MAGI1*-variant positive	and	High MB	0 (0)	7 (70)	0.003

Values are expressed as n (%).

### The frequencies of the specific gene variants in TCGA cohort

We analyzed these identified markers using the variant data sets of gastric adenocarcinomas (n = 440) in the TCGA cohort^15^, although there were no available variant data of non-cancerous mucosa with information on metachronous development. In the TCGA cohort, gastric cancer (GC) harboring variants in *RECQL4*, *ARID1A*, *MAGI1*, or *JAK3* were 4.5% (n = 20), 27.0% (n = 119), 7.5% (n = 33), or 3.8% (n = 17), respectively. Of *RECQL4*-variants positive GCs, which is the criteria of the initial EGC for predicting non-MGC development in the present study, 70% of the GCs were negative for the variants in both *ARID1A* and *MAGI1*. Of both *ARID1A*- and *MAGI1*-variants positive GCs (n = 24, 5.4%), which is the criteria of initial EGC for predicting MGC development in the present study, 75% of the GCs were negative for the variants in *RECQL4*. These criteria were seldom met in the same GCs in the TCGA cohort (1.3%, *p* < 0.01). These findings suggest that each criteria of the initial EGC in the present study seems to indicate a different genomic characteristic group of GC development.

## Discussion

Molecular analyses from large-scale, international, collaborative studies have elucidated the genomic landscape of gastric cancer, highlighting its enormous heterogeneity and the complex interplay between genetic and epigenetic alterations^14–16^. This has allowed identification of biomarkers with predictive, therapeutic, and prognostic values that may help physicians offer more personalized medicine than possible before. A recent study showed the genomic characteristics of both primary advanced GC and lymph nodes metastasis by using whole-exome sequencing^17^. However, EGC’s molecular features remain unclear. In this present study, we evaluated somatic variants of 409 cancer-related genes and MBs in both EGC and adjacent non-cancerous mucosal specimens obtained from patients who later developed metachronous cancers and from those who remained in remission. We found that somatic genomic alterations of the *RECQL4*, *JAK3*, *ARID1A*, and *MAGI1* genes, combined with MBs, were associated with the development of metachronous cancers after curative ESD and successful HP eradiation. These findings provide new evidence of molecular EGC stratification to predict MGC and should help develop a specific follow-up strategy as well as elucidate the mechanisms.

HP has been accepted as the most important risk factor for gastric carcinogenesis^18,19^, and its eradication should have prevented metachronous developments^10^; however, the risk of MGC cannot be removed completely. In this study, we revealed different genomic features of the initial EGCs and their adjacent non-cancerous mucosas in specimens from MGC patients (M group) and in those without MGC developments (N group), although both groups had similar clinical and endoscopic characteristics. These findings suggest that HP-infected individuals harbor different genomic alterations during early cancer stages. The timing of HP eradiation affects not only the eradication rate, but also the MGC incidence rate^10,20^. Our findings may be associated with a point-of-no-return in gastric carcinogenesis. Further studies are required to clarify the factors leading to these somatic alterations (such as host genomic factors, HP strains, and its virulence factors).

A high tumor mutational burden (TMB) is an emerging biomarker of sensitivity to immune checkpoint inhibitors^21^. Target capture sequencing may identify actionable genes and further determine tumor or mucosal MBs in a more cost-effective, efficient manner that will achieve higher sequencing depths than whole exome sequencing. A study using a hybridization, capture-based NGS panel, Biotecan PanCancer Panoramic Detection, revealed that the tumor mutational burden is associated with DNA damage repair genes, Lauren classification, differentiation, and HER2 status^22^. In that study, more than 80% of subjects had advanced GCs, and data of non-cancerous mucosa were not shown. In our study, we found high MBs in the adjacent non-cancerous mucosa in the specimens from the M group, despite the fact that the TMBs in the initial EGC specimens were similar in both groups. We speculate that a highly mutated gastric mucosa may reflect a cancerous background that may lead to development of MGCs. MB is a potential predictive marker for MGC and plays an important role in its pathogenesis.

In this study, somatic alterations of two cancer-related genes, including the *RECQL4* and the *JAK3,* had significant associations with non-MGC development, and the *ARID1A* and the *MAGI1* genes had significant associations with metachronous development. RECQL4 belongs to the RecQ helicase family, a group of DNA unwinding enzymes that ensure proper repair of DNA damage to maintain genome stability, replication, recombination, and transcription^23^. Mutational loss of the *RECQL4* is associated with increased risk for osteosarcoma development in Rothmund-Thomson syndrome^24^. Studies have demonstrated that RECQL4 acts as a tumor promotor in some cancers, such as prostate^25^, breast^26^, and colorectal cancers^27^. Only two other studies have investigated associations between the RECQL4 and GC. One reported that RECQL4 drives cisplatin resistance by activating an AKT-YB1-MDR1 signaling pathway^28^. The other study showed that increased RECQL4 expression may predict poor prognosis in GC patients^29^. In GC patients, *RECQL4* seems to be associated with treatment resistance, and EGC harboring the *RECQL4* variant was associated with non-MGC development. JAK3 is a protein-tyrosine kinase of the JAK family composed of four members, including JAK1, JAK2, JAK3 and tyrosine kinase 2 (TYK2). JAK1, JAK2, and TYK2 are thought to be expressed ubiquitously, whereas JAK3 is confined to hematopoietic, myeloid, and lymphoid cells^30^. JAK3, a potential tumorigenic-regulator, was shown to be upregulated directly by activation of signal transducer and activator of transcription 3 (STAT3) following HP infection^31^. Further studies will need to clarify *RECQL4* and *JAK3* variants’ roles in GC as well as in infiltrating inflammatory cells.

The *ARID1A* gene encodes a key component of the adenosine triphosphate-dependent chromatin-modeling complex switch/sucrose-nonfermentable (SWI/SNF) chromatin-remodeling complex discovered to be a driver for ovarian clear cell carcinoma (as a tumor-suppressor)^32,33^. Exome sequencing analyses in GC identified frequent inactivating mutations or protein deficiency of *ARID1A* in 83% of cases with microsatellite instability (MSI)^34^ and an association with concurrent *PIK3CA* mutations and MSI^35^. ARID1A inactivation leads to an altered mismatch repair, and increased mutagenesis, and its deficiency correlates with MSI^36^. In our study, we revealed that an EGC harboring a variant of the *ARID1A,* with a high MB in the adjacent non-cancerous mucosa, was associated with an increased rate of developing MGC after HP eradication. Moreover, the methylation accumulation in non-cancerous gastric mucosa of GC patients has been suggested to be a promising biomarker for risk of MGC development^12,13^. These findings suggest that disordered chromatin-remodeling in a genomic unstable gastric mucosa may lead to gastric carcinogenesis and may be associated with difficulties in reducing MGC risk after HP eradication. In addition to *ARID1A* alterations, the environment inducing *MAGI1* alterations during the EGC may be a MGC risk factor. MAGI1 is a member of the MAGI subfamily of membrane associated guanylate kinases, which is emerging as important in coupling the extracellular environment to intracellular signaling pathways and the cytoskeleton at synapses and tight junctions^37^. The interaction between HP CagA and the partitioning-defective 1 (PAR1) disrupts tight junctions and causes loss of epithelial cell polarity^38^. Reports have demonstrated that MAGI1 acts as a tumor suppressor by inhibiting the mitogen-activated protein kinase/extracellular signal-regulated kinase signaling pathway in GC^39^. The MAGI1 modulation, based on a disorganized tight junction, may underlie HP-infection induced gastric carcinogenesis.

We are aware of the limitations of our study. First, a selection bias may exist due to retrospective sample collection. Second, the limited sample size could affect the conclusion. Future studies with larger populations are required to verify our results. It is important to evaluate the quality of our findings in this discovery cohort using the new setting validation cohort. Third, further investigations need to determine the molecular mechanisms behind the association between somatic alterations or MB and development of MGC. Different gastric tumors possess multiple clonal origins^40^. We did not compare the genomic characteristics of MGC and the initial EGC, which would have been interesting.

In conclusion, we presented that the combined assessment of specific somatic genomic alterations and the MB of the non-cancerous mucosa may differ in the M and N groups. These differences may help to identify the predictive biomarkers for developing MGC after curative ESD and successful HP eradiation. This study included a limited number of subjects and needs to be validated in larger studies; however, our findings might provide new insights into understanding the genomic landscape and mutational profile underlying EGC development, and have potential significant implications for improving future screening or treatment strategies for gastric cancer.

## Methods

### Participants

We retrospectively screened 283 EGC patients who underwent ESD at Yamagata University Hospital between January 2009 and December 2012. Among them, we selected 10 patients who had developed MGC more than a year after undergoing curative resection and successful HP eradication (M group). We then selected ten patients with no MGC developments for more than 3 years after ESD and successful HP eradication (N group), ensuring they matched the other 10 patients in M group in terms of age, gender, and location, size, and histological type of the initial EGC (Fig. 1A). We considered resections as curative when all the following pathological conditions were fulfilled: en bloc resection of the intestinal histological type, mucosal lesion, negative horizontal margin, negative vertical margin, and no lympho-vascular infiltration^41^. This assessment enabled us to distinguish between the part of cancer and adjacent non-cancerous tissue in the tissue specimen. We obtained clinical information on height, weight, alcohol consumption, smoking, and endoscopic findings of participants from their medical records.

The Ethics Committee of Yamagata University Faculty of Medicine approved this study (#2017-490), and we conducted it in accordance with the tenets of the Declaration of Helsinki. Written informed consent was obtained from all subjects.

### Cancer-related gene panel sequencing

We extracted DNA separately, using the GeneRead DNA FFPE tissue kit (QIAGEN, Hilden, Germany), from the initial 10% formalin-fixed paraffin-embedded ESD-resected EGC and adjacent non-cancerous mucosa specimens. We used AmpliSeq Library Kit v2.0 (Thermo Fisher Scientific, Waltham, MA, USA) to construct libraries according to the manufacturer’s instructions. Quantification of the libraries was performed on a 2200 TapeStation System using High Sensitivity D1000 Reagents and High Sensitivity D1000 ScreenTape (Agilent Technologies, Santa Clara, CA, USA). We submitted amplified libraries to emulsion PCR, using an Ion OneTouch 2 System with an Ion PI Template OT2 200 Kit v3 (Thermo Fisher Scientific, Waltham, MA, USA). Ion sphere particles were enriched, using Ion OneTouch ES, and were loaded onto an Ion PI Chip v2. NGS was performed by Ion Proton with the Ion Proton and Ion AmpliSeq Comprehensive Cancer Panel (Thermo Fisher Scientific, Waltham, MA, USA), which targeted 409 genes (Suppl. Table 5). Read sequence files were run through the assembly programs constructed with Bowtie2 (http://bowtie-bio.sourceforge.net/bowtie2/index.shtml) and BWA (http://bio-bwa.sourceforge.net/bwa.shtml), while consecutively carrying out a Freebayes program (https://github.com/ekg/freebayes) to obtain variant call format files. Concordant genetic variants, detected by the two pipelines, were annotated using ANNOVAR (http://annovar.openbioinformatics.org).

### Somatic variant analysis

After excluding synonymous mutations, we extracted the variants with a minor allele frequency not reported in the following genome databases: 1000 Genomes Project (https://www.internationalgenome.org/home), ExAC (http://exac.broadinstitute.org/), and our own healthy general population (N = 176) cohort databases^42^. We defined the detected variants as somatic variants in this study. The somatic variants in the initial EGC and adjacent non-cancerous mucosa were compared between primary ESD-resected specimens obtained from the M and N groups (Fig. 1B).

### Mutational burden assessments

The number of somatic variants detected on NGS, using the Ion AmpliSeq Comprehensive Cancer Panel (Thermo Fisher Scientific, Waltham, MA, USA), interrogated 1.29 Mb of the genome. We calculated the MB in variants per megabase (Mb) using maftools^43^. Based on the ROC analysis to determine the MB cutoff point to predict MGC developments, we divided the MB levels into one of two groups: low (less than 7.75 variants/Mb) and high (≥ 7.75 variants/Mb).

### Statistical analyses

We analyzed continuous variables and categorical variables using the two-tailed Wilcoxon test and the Fisher exact test, respectively. We used the Kruskal–Wallis test to compare continuous variables following a post-hoc Steel–Dwass test. We computed the ORs and 95% confidence intervals using logistic regression model analyses. We considered differences with *P* < 0.05 to be statistically significant. We carried out statistical calculations using JMP 14 (SAS Institute) and R programming language version 3.6.1 software.

## Supplementary Information

Supplementary Information.

Supplementary Tables.

## Data Availability

All data generated or analyzed during this study are included in this article. All other data generated and/or analyzed to support our findings are available from the corresponding author upon reasonable request.

## References

[1] Bray F (2018). Global cancer statistics 2018: GLOBOCAN estimates of incidence and mortality worldwide for 36 cancers in 185 countries. CA Cancer J. Clin..

[2] Hasuike N (2018). A non-randomized confirmatory trial of an expanded indication for endoscopic submucosal dissection for intestinal-type gastric cancer (cT1a): The Japan Clinical Oncology Group study (JCOG0607). Gastric Cancer.

[3] Ono H (2016). Guidelines for endoscopic submucosal dissection and endoscopic mucosal resection for early gastric cancer. Dig. Endosc..

[4] Kato M (2019). Metachronous gastric cancer risk after endoscopic resection of early gastric cancer and *H. pylori* status. J. Gastroenterol..

[5] Abe S (2018). Metachronous gastric cancer following curative endoscopic resection of early gastric cancer. Clin. Endosc..

[6] Fukase K (2008). Effect of eradication of *Helicobacter**pylori* on incidence of metachronous gastric carcinoma after endoscopic resection of early gastric cancer: An open-label, randomised controlled trial. Lancet.

[7] Choi JM (2018). Effects of *Helicobacter**pylori* eradication for metachronous gastric cancer prevention: A randomized controlled trial. Gastrointest. Endosc..

[8] Choi IJ (2018). *Helicobacter**pylori* therapy for the prevention of metachronous gastric cancer. N. Engl. J. Med..

[9] Mori G (2016). Incidence of and risk factors for metachronous gastric cancer after endoscopic resection and successful *Helicobacter**pylori* eradication: results of a large-scale, multicenter cohort study in Japan. Gastric Cancer.

[10] Xiao S, Li S, Zhou L, Jiang W, Liu J (2019). *Helicobacter**pylori* status and risks of metachronous recurrence after endoscopic resection of early gastric cancer: A systematic review and meta-analysis. J. Gastroenterol..

[11] Hirata K (2013). CD44 variant 9 expression in primary early gastric cancer as a predictive marker for recurrence. Br. J. Cancer.

[12] Asada K (2015). Demonstration of the usefulness of epigenetic cancer risk prediction by a multicentre prospective cohort study. Gut.

[13] Maeda M (2017). High impact of methylation accumulation on metachronous gastric cancer: 5-year follow-up of a multicentre prospective cohort study. Gut.

[14] Tan P, Yeoh K-G (2015). Genetics and molecular pathogenesis of gastric adenocarcinoma. Gastroenterology.

[15] Cancer T (2014). Comprehensive molecular characterization of gastric adenocarcinoma. Nature.

[16] Jácome AA, Coutinho AK, Lima EM, Andrade AC, Dos Santos JS (2016). Personalized medicine in gastric cancer: Where are we and where are we going?. World J. Gastroenterol..

[17] Zhang J (2018). Genomic alterations in gastric cancers discovered via whole-exome sequencing. BMC Cancer.

[18] Amieva M, Peek RM (2016). Pathobiology of *Helicobacter**pylori*-induced gastric cancer. Gastroenterology.

[19] Uemura N (2001). *Helicobacter**pylori* infection and the development of gastric cancer. N. Engl. J. Med..

[20] Sokic-Milutinovic A, Alempijevic T, Milosavljevic T (2015). Role of *Helicobacter**pylori* infection in gastric carcinogenesis: Current knowledge and future directions. World J. Gastroenterol..

[21] Chalmers ZR (2017). Analysis of 100,000 human cancer genomes reveals the landscape of tumor mutational burden. Genome Med..

[22] Cai H (2019). Mutational landscape of gastric cancer and clinical application of genomic profiling based on target next-generation sequencing. J. Transl. Med..

[23] Bernstein KA, Gangloff S, Rothstein R (2010). The RecQ DNA helicases in DNA repair. Annu. Rev. Genet..

[24] Wang LL (2003). Association between osteosarcoma and deleterious mutations in the RECQL4 gene in Rothmund–Thomson syndrome. J. Natl. Cancer Inst..

[25] Su Y (2010). Human RecQL4 helicase plays critical roles in prostate carcinogenesis. Cancer Res..

[26] Fang H (2013). RecQL4 helicase amplification is involved in human breast tumorigenesis. PLoS ONE.

[27] Lao VV (2013). Altered RECQ helicase expression in sporadic primary colorectal cancers. Transl. Oncol..

[28] Mo D (2016). Human helicase RECQL4 drives cisplatin resistance in gastric cancer by activating an AKT-YB1-MDR1 signaling pathway. Cancer Res..

[29] Chen H (2018). Overexpression of RECQL4 is associated with poor prognosis in patients with gastric cancer. Oncol. Lett..

[30] Roskoski R (2016). Janus kinase (JAK) inhibitors in the treatment of inflammatory and neoplastic diseases. Pharmacol. Res..

[31] Zhao J (2014). *Helicobacter**pylori*-induced STAT3 activation and signalling network in gastric cancer. Oncoscience.

[32] Jones S (2010). Frequent mutations of chromatin remodeling gene ARID1A in ovarian clear cell carcinoma. Science.

[33] Wiegand KC (2010). ARID1A mutations in endometriosis-associated ovarian carcinomas. N. Engl. J. Med..

[34] Wang K (2011). Exome sequencing identifies frequent mutation of ARID1A in molecular subtypes of gastric cancer. Nat. Genet..

[35] Zang ZJ (2012). Exome sequencing of gastric adenocarcinoma identifies recurrent somatic mutations in cell adhesion and chromatin remodeling genes. Nat. Genet..

[36] Shen J (2018). ARID1A deficiency promotes mutability and potentiates therapeutic antitumor immunity unleashed by immune checkpoint blockade. Nat. Med..

[37] Feng X, Jia S, Martin TA, Jiang WG (2014). Regulation and involvement in cancer and pathological conditions of MAGI1, a tight junction protein. Anticancer Res..

[38] Saadat I (2007). Helicobacter pylori CagA targets PAR1/MARK kinase to disrupt epithelial cell polarity. Nature.

[39] Jia S (2017). MAGI1 inhibits migration and invasion via blocking MAPK/ERK signaling pathway in gastric cancer. Chin. J. Cancer Res..

[40] Takaoka S (2019). Molecular subtype switching in early-stage gastric cancers with multiple occurrences. J. Gastroenterol..

[41] Sano T, Kodera Y (2017). Japanese gastric cancer treatment guidelines 2014 (ver. 4). Gastric Cancer.

[42] Koyama S (2017). Clinical and radiological diversity in genetically confirmed primary familial brain calcification. Sci. Rep..

[43] Mayakonda A, Lin DC, Assenov Y, Plass C, Koeffler HP (2018). Maftools: Efficient and comprehensive analysis of somatic variants in cancer. Genome Res..

