# Supplementary material for: Somatic alterations and mutational burden are potential predictive factors for metachronous development of early gastric cancer
Source: Sci Rep. 2020 Dec 16;10:22071. doi: 10.1038/s41598-020-79195-0 (PMC7745026; doi:10.1038/s41598-020-79195-0)
Supplement: Supplementary file 1 — Supplementary Information. [file 41598_2020_79195_MOESM1_ESM.docx]

**Somatic alterations and mutational burden are potential predictive factors for metachronous development of early gastric cancer**

Kazuhiro Sakuta^1^, Yu Sasaki^1, *^, Yasuhiko Abe^1,2^, Hidenori Sato^3^, Masakuni Shoji^1^, Takao Yaoita^1^, Makoto Yagi^1,2^, Naoko Mizumoto^1^, Yusuke Onozato^1^, Takashi Kon1, Ayumi Koseki^1^, Sonoko Sato^1^, Ryoko Murakami^3^, Yuki Miyano^3^, and Yoshiyuki Ueno^1^

^1^Yamagata University Faculty of Medicine, Department of Gastroenterology, 2-2-2 Iida-Nishi, Yamagata-city, 9909585, Japan

^2^Yamagata University Hospital, Division of Endoscopy, 2-2-2 Iida-Nishi, Yamagata-city, 9909585, Japan

^3^Yamagata University Faculty of Medicine, Department of Genomic Cohort Research, Genomic Information analysis Unit, 2-2-2 Iida-Nishi, Yamagata-city, 9909585, Japan

***Corresponding author**:

Yu Sasaki

Department of Gastroenterology, Faculty of Medicine, Yamagata University,

2-2-2 Iida-Nishi, Yamagata 990-9585, Japan

E-mail: y-sasaki@med.id.yamagata-u.ac.jp

Tel: +81-23-628-5309

Fax: +81-23-628-5311

**Supplementary information accompanying this paper**

**Supplementary figure legends**

**Supplementary Figure 1**. Graph of variant load against TCGA cohorts


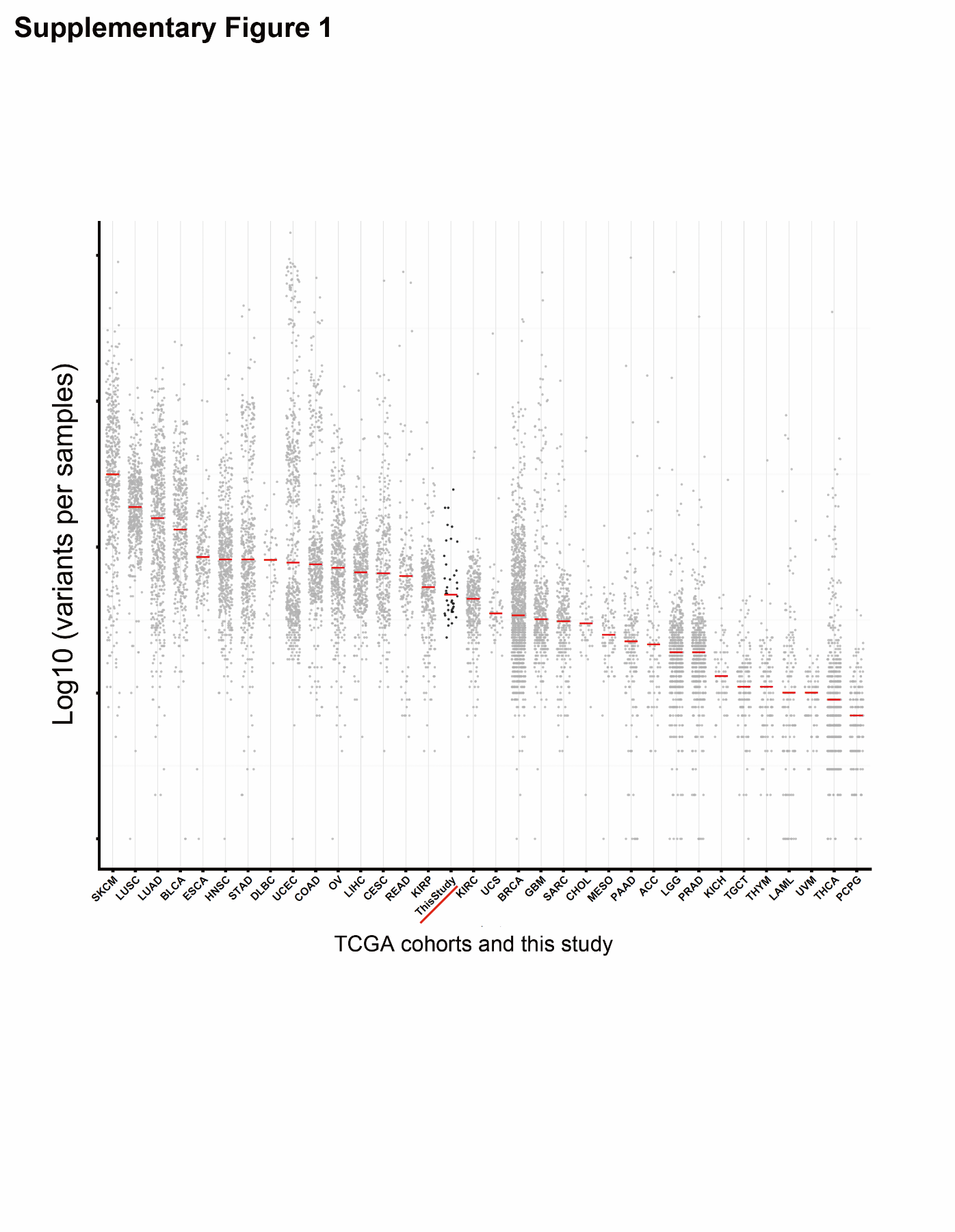


Every dot represents a sample; the red horizontal lines are the median numbers of variants in the respective cancer types. The vertical axis (log scale) shows the number of variants per sample. The different cancer types are displayed on the horizontal axis based on their median numbers of somatic variants. The variant load in this study stands almost in the middle of the 33 landmark cohorts in the Cancer Genome Atlas (TCGA). SKCM, skin cutaneous melanoma; LUSC, lung squamous cell carcinoma; LUAD, lung adenocarcinoma; BLCA, bladder urothelial carcinoma; ESCA, esophageal carcinoma; HNSC, head and neck squamous cell carcinoma; STAD, stomach adenocarcinoma; DLBC, diffuse large B-cell lymphoma; UCEC, uterine corpus endometrial carcinoma; COAD, colon adenocarcinoma; OV, ovarian serous cystadenocarcinoma; LIHC, liver hepatocellular carcinoma; CESC, cervical squamous cell carcinoma and endocervical adenocarcinoma; READ, rectum adenocarcinoma; KIRP, kidney renal papillary cell carcinoma; KIRC, kidney renal clear cell carcinoma; UCS, uterine carcinosarcoma; BRCA, breast invasive carcinoma; GBM, glioblastoma multiforme; SARC, sarcoma; CHOL, cholangiocarcinoma; MESO, mesothelioma; PAAD, pancreatic adenocarcinoma; ACC, adrenocortical carcinoma; LGG, brain lower grade glioma; PRAD, prostate adenocarcinoma; KICH, kidney chromophobe; TGCT, testicular germ cell tumors; THYM, thymoma; LAML, acute myeloid leukemia; UVM, uveal melanoma; THCA, thyroid carcinoma; PCPG, pheochromocytoma and paraganglioma.

**Supplementary Figure 2**. Transitions and Transversions


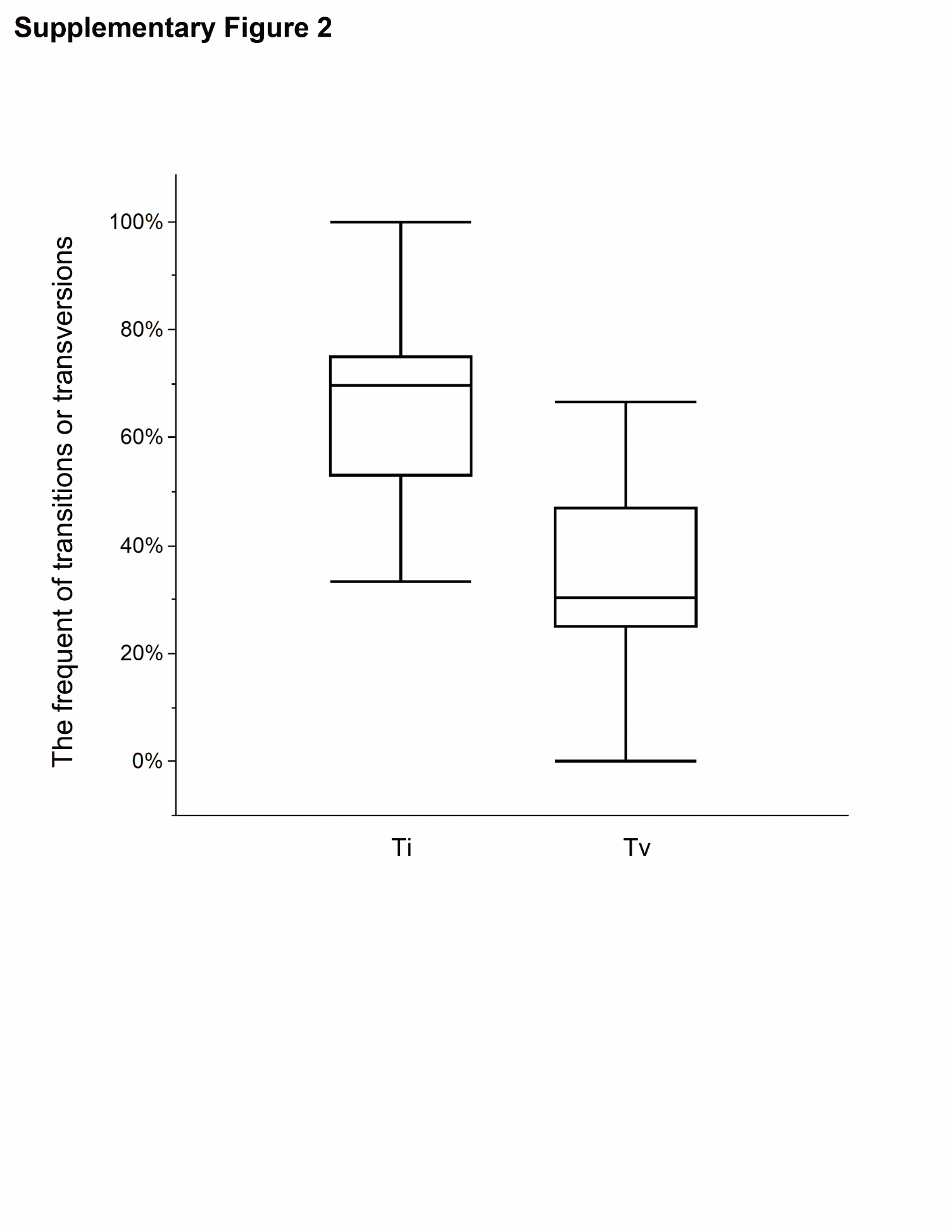


The median number of transition (Ti) or transversion (Tv) SNPs were 14.5 [interquartile range (IQR), 7.0–31.0] and 9 (IQR, 6.0–13.0), respectively. The Ti or Tv frequencies were 69.6% (IQR, 52.9–75.0) or 30.3% (IQR, 25.0–47.0), respectively. The median Ti/Tv ratio was 2.25 (IQR, 1.11–3.00).

**The following Supplementary Tables 1 to 5 are provided as Excel files**:

**Supplementary Table 1**. Characteristics of the patients

**Supplementary Table 2**. Complete list of all somatic variants

**Supplementary Table 3**. Comparison of somatic variants among the groups

**Supplementary Table 4**. ROC table

**Supplementary Table 5**. Target genes in Ion AmpliSeq Comprehensive Cancer Panel
